# Occurrence of Sex Chromosomes in Fish of the Genus *Ancistrus* with a New Description of Multiple Sex Chromosomes in the Ecuadorian Endemic *Ancistrus clementinae* (Loricariidae)

**DOI:** 10.3390/genes14020306

**Published:** 2023-01-24

**Authors:** Mauro Nirchio, Claudio Oliveira, Marcelo de Bello Cioffi, Francisco de Menezes Cavalcante Sassi, Jonathan Valdiviezo, Fabilene Gomes Paim, Leticia Batista Soares, Anna Rita Rossi

**Affiliations:** 1 Departamento de Acuacultura, Universidad Técnica de Machala, Av. Panamericana km 5.5, Vía Pasaje, Machala 070150, Ecuador; 2 Departamento de Morfologia, Instituto de Biociências Universidade Estadual Paulista-UNESP, Botucatu 18618-689, SP, Brazil; 3 Departamento de Genética e Evolução, Universidade Federal de São Carlos, São Carlos 13565-090, SP, Brazil; 4 Instituto Nacional de Biodiversidad, Rumipamba No. 341 y Av. Shyris, Parque La Carolina, Quito 170506, Ecuador; 5 Dipartimento di Biologia e Biotecnologie “C. Darwin”, Sapienza-Università di Roma, Via Alfonso Borelli 50, 00161 Rome, Italy

**Keywords:** ZZ/ZW_1_W_2_ sex system, teleost, rDNA, telomeric repeats, Hypostominae, Ancistrini

## Abstract

*Ancistrus* Kner, 1854, is the most diverse genus among the Ancistrini (Loricariidae) with 70 valid species showing a wide geographic distribution and great taxonomic and systematic complexity. To date, about 40 *Ancistrus* taxa have been karyotyped, all from Brazil and Argentina, but the statistic is uncertain because 30 of these reports deal with samples that have not yet been identified at the species level. This study provides the first cytogenetic description of the bristlenose catfish, *Ancistrus clementinae* Rendahl, 1937, a species endemic to Ecuador, aiming to verify whether a sex chromosome system is identifiable in the species and, if so, which, and if its differentiation is associated with the presence of repetitive sequences reported for other species of the family. We associated the karyotype analysis with the COI molecular identification of the specimens. Karyotype analysis suggested the presence of a ♂ZZ/♀ZW_1_W_2_ sex chromosome system, never detected before in *Ancistrus,* with both W_1_W_2_ chromosomes enriched with heterochromatic blocks and 18S rDNA, in addition to GC-rich repeats (W_2_). No differences were observed between males and females in the distribution of 5S rDNA or telomeric repeats. Cytogenetic data here obtained confirm the huge karyotype diversity of *Ancistrus,* both in chromosome number and sex-determination systems.

## 1. Introduction

According to Eschmeyer’s Catalog of Fishes [1], the Loricariidae (Siluriformes) family of fish is one of the largest in the world with 1029 species that are found across the Neotropics and are adapted to a variety of environments, including fresh and brackish waters [2,3,4]. Members of this taxon are popularly known as plecos, suckermouth catfishes or armored catfishes, since they present a body without scales and are fully covered with ossified plates, together with a ventral-positioned suckermouth This catfish family is divided into six subfamilies (Lithogeninae, Delturinae, Rhinelepinae, Loricariinae, Hypoptopomatinae and Hypostominae) [5]; among these, the subfamily Hypostominae ranks first in species number containing 498 valid species and 45 genera [1] grouped into five tribes (Ancistrini, Corymbophanini, Hypostomini, Pterygoplichthini, and Rhinelepini) [6]. The genus *Ancistrus* Kner, 1854 (tribe Ancistrini) is the most species-rich among Ancistrini with 70 valid species of small fishes, some showing brilliant colors, that are exploited in the ornamental fish trade [7]. *Ancistrus* is characterized by poorly resolved alpha taxonomy [8] and shows wide geographic distribution [9] and ecological diversity, including species that inhabit aquatic systems from both trans– and cis–Andean regions, from Panama to Argentina, in lentic lowland waters and torrential mountain streams [8,10,11].

Four *Ancistrus* species were reported in Ecuador, three of which live in freshwater systems that drain into the Amazon River Basin, i.e., *A. alga* (Cope, 1872), inhabiting the northern and central portions of the eastern area of the country; *A. malacops* (Cope, 1872), with broad distribution from north to south, but absent from the Santiago River; *A. shuar* Provenzano and Barriga-Salazar, 2018, restricted to the Santiago River, in Morona-Santiago province. The fourth species, *A. clementinae* Rendahl, 1937, is endemic to Ecuador and is found in Pacific slope aquatic systems, primarily in the Guayas River drainage [12]. This latter species, commonly known as the bristlenose cleaner catfish, is in high demand in the aquarium trade, despite not having any immediate economic importance [13], because it feeds on both algae and debris that are found at the aquarium’s bottom (food leftovers, but also remains of fish or dead fish) [14].

Loricariidae and Ancistrini catfishes were only partially karyologically analyzed, despite fish cytogenetics being known to provide useful information on the differentiation of related taxa, allowing the detection of cryptic species and the identification of sex-associated sequences and heterochromosomes [15]. Indeed, only 74 species of this family are reported in the most comprehensive checklist of fish karyotypes [16], 24 of which belong to the genus *Ancistrus*. This number was raised to 43 in later investigations, although this is not a certain estimate as most of the reports refer to samples that were not identified at the species level and are reported as *Ancistrus* sp. or are considered synonyms (see [17]). These data revealed both taxonomic uncertainty and high karyotype diversity among *Ancistrus* species from Brazil and Argentina, with a diploid number ranging from 2n = 34 to 54 chromosomes, and the presence of species without cytologically identified sex chromosomes, and others with standard (XX/XY, ZZ/ZW) or multiple sex chromosome (MSC) systems, such as XX/XY_1_Y_2_, and Z_1_Z_1_Z_2_Z_2_/Z_1_Z_2_W_1_W_2_ [2,7,16,17,18,19,20,21,22,23,24]. To date, species from Ecuador have not been analyzed.

In this research, we provide the first cytogenetic description of *A. clementinae* Rendahl, 1937, using conventional (Giemsa staining, C-banding and silver staining) and molecular (fluorescence in situ hybridization—FISH) cytogenetic methods. The cytogenetic analysis was associated with the molecular identification of the specimens by mitochondrial sequence analysis. The study aims at: (a) verifying whether, cytologically, sex chromosomes are identifiable and which sex chromosome system is present in the species, and whether its differentiation is associated with the presence of repetitive sequences, as observed in other *Ancistrus* from Brazil [22]; (b) verifying whether this karyotype shows plesiomorphic characteristics or can be considered derived (i.e. showing a reduced diploid chromosome number) compared to those of the species present in river systems from the cis-Andean region that flow into the Atlantic Ocean and/or belonging to different basins; (c) anchoring the new karyotype to a certainly identified species, on morphological and molecular bases.

## 2. Materials and Methods

### 2.1. Sampling and Morphological Identification

**Figure 1:**
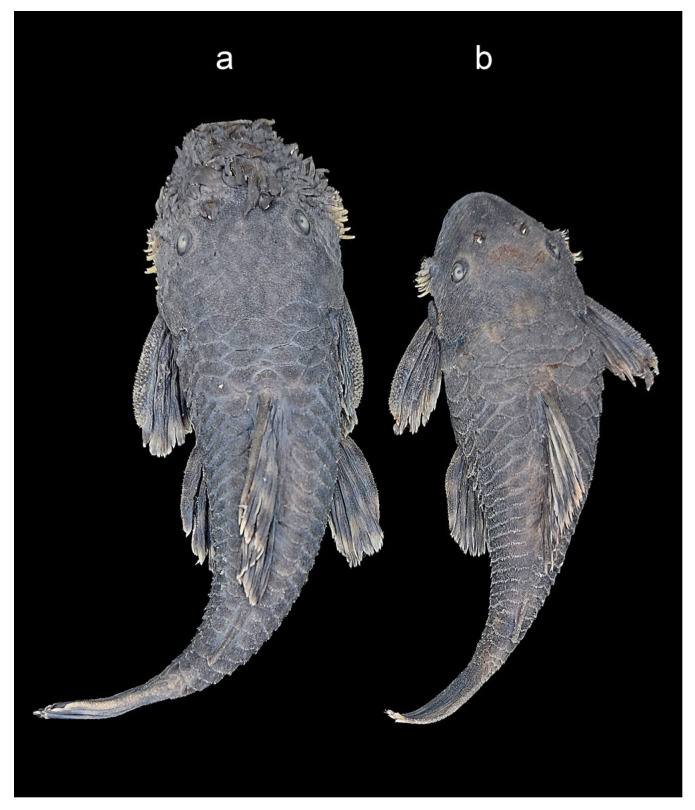
Male (**a**) and female (**b**) specimens of *A. clementinae*.

A sample composed of 24 specimens of *A. clementinae* Rendahl, 1937, (10 males and 14 females) collected with a seine net in Río Palenque (Cantón Pasaje) and Río La Moquillada (Cantón Las Lajas) El Oro Province, Ecuador, were analyzed (Table S1). Morphological specimen identification was performed following Provenzano and Barriga-Salazar [12]. Males showed soft, fleshy tentacles on the snout and two backward divergent rows of flat-branched tentacles, absent in females (see Figure 1). The fishes were transported alive to the laboratory in sealed plastic bags (32 inches) containing two gallons of water, replacing the air with pure oxygen, and finally kept in aquariums until they were processed.

Voucher specimens (Figure 1) were deposited in the ichthyological collection of the Instituto Nacional de Biodiversidad (INABIO) of Ecuador (MECN-DP 4958, MECN-DP 4961). 

### 2.2. Molecular Identification of Samples and Phylogenetic Reconstruction

The Wizard Genomic DNA Purification kit (Promega, Madison, WI, USA) was used for extracting genomic DNA from muscle tissue following the manufacturer’s instructions. The partial sequence of the mitochondrial cytochrome oxidase I (COI) gene was amplified using polymerase chain reaction (PCR) using the Fish F1 and Fish R1 primers [25]. PCR reactions were carried out in an Applied Biosystems thermocycler (Applied Biosystems, Foster City, CA, USA) following protocols reported by [26]. The BigDye sequencing kit cycle terminators (Applied Biosystems, Foster City, CA, USA) were used as reagent components in all sequencing reactions, according to the manufacturer’s instructions, and sequences were analyzed using an ABI PRISM 3130 Genetic Analyzer (Applied Biosystems, Foster City, CA, USA).

Sequences were aligned using the software Clustal X. [27]. The basic local alignment search tool (BLAST, https://blast.ncbi.nlm.nih.gov/Blast/, accessed on 30 November 2022) was used to search the GenBank database for similar sequences, and the BOLD system (https://www.boldsystems.org/, accessed on 30 November 2022) was also explored for reference sequences. Sequences of the other *Ancistrus* species available in the GenBank database were retrieved and included in the analysis (at least two sequences for each species, when available; *Ancistrus* sp. records were excluded) and *Lithoxus stocki* was used as an outgroup (Table S2).

Phylogenetic reconstruction was obtained from the data set with neighbor-joining (NJ) analyses using 1000 bootstrap pseudoreplicates; this reconstruction and the genetic distances calculation were performed using MEGA5 [28]. 

### 2.3. Cytogenetic Procedures

Each fish was stimulated to increase the number of metaphases with an injection of yeast suspension [29] in the caudal peduncle 48 h before injecting intra-abdominally a dose of 0.01 mL/g colchicine (0.0125%). After colchicine treatment, fish were maintained at room temperature for 24–48 h in a well-aerated aquarium and finally euthanized with an overdose of benzocaine [30].

Cell suspensions containing mitotic chromosomes were obtained from the animals’ kidneys [31]. Chromosomes were stained with 5% Giemsa solution (phosphate buffer, pH 6.8) to define the diploid numbers (2n) and karyotype formula. Heterochromatic regions were identified by the C-banding procedure [32]. Nucleolus organizer regions (NORs) were stained following silver nitrate impregnation [33].

The fluorescence in situ hybridization experiments were performed according to Pinkel et al. [34], with some adaptations described in Soares et al. [35] and Sassi et al. [36]. In summary, metaphase chromosomes were treated with RNAse A (40 μg/mL) for 1.5 h at 37 °C and denatured in 70% formamide/2× SSC at 72 °C for 3 min. The hybridization mixture (2.5 ng/μL probes, 50% deionized formamide, 10% dextran sulfate) was applied to the slides and hybridization was performed for 14 h at 37 °C in a dark moist chamber. The probes of major and minor ribosomal genes (18S rDNA, 5S rDNA), and telomeric sequence (TTAGGG)n were obtained by polymerase chain reaction (PCR) using primers described by Utsunomia et al. [37], Pendas et al. [38], and Ijdo et al. [39], respectively. These probes were directly labeled through Nick-Translation, using Atto488-dUTP (18S rDNA) or Atto550-dUTP (5S rDNA and telomeric sequence), according to the manufacturer’s instructions (Jena Biosciences, Jena, Germany). Chromosomes were counterstained with 4′,6-diamidino-2-phenylindole (DAPI) and mounted in antifading solution (Vector Laboratories, Burlington, ON, Canada) after a post-hybridization wash in 1× SSC at 65 °C and 4× SSC/Tween at room temperature for 5 min each.

For each individual, at least 60 metaphases were recorded to determine the diploid modal number and confirm the FISH results, from which the best 20 to 30 mitotic figures were chosen to acquire photographs.

### 2.4. Images Capture and Processing

All images were recorded with an Olympus BX53 epifluorescence microscope (Olympus Corporation, Ishikawa, Japan) equipped with an Olympus DP73 digital camera coupled to cellSens Dimension Software (Olympus) for image acquisition. Images were merged and edited for optimization of brightness and contrast using Photoshop (Adobe Systems, Inc., San José, CA, USA, Version 2015.0.0). Chromosomes were arranged in decreasing order and identified according to arm ratio criteria as metacentric (m), submetacentric (sm), subtelocentric (st) and acrocentric (a) [40]. The chromosome arms number (fundamental number, FN) was established considering subtelocentric and acrocentric chromosomes as uni-armed and m-sm elements as bi-armed.

## 3. Results

### 3.1. Molecular Identification of Samples and Phylogenetic Reconstruction

Sequences obtained in this study for *A*. *clementinae* COI (651 base pairs) were deposited in GenBank (A.N. OQ132522-23). No other COI sequence was present in the system for this species, and BLAST function gave back the highest similarities (88–89%) with sequences belonging to *A. aguaboensis*, *A. cirrhosis*, *A. cryptophthalmus* and *Ancistrus* sp. 

The phylogenetic reconstructions (Figure S1) obtained by the inclusion of all species-level identified *Ancistrus* sequences available in GenBank showed that our samples form a monophyletic well-supported and homogeneous clade within the genus. 

These results were confirmed by the BOLD database, where no match was found for our sequences, and where they clustered within *Ancistrus* (in the BOLD tree that includes also private records), not intermixed with any other species of the genus and close to the group consisting of the Colombian *A. caucanu.*

The genetic distances (Kimura 2-parameters distance, K2P) [41] between *A. clementinae* and the other *Ancistrus* species ranged from 12.44% to 16.77%, obtained with *A._cryptophthalmus* and *A._cf. leucostictus*, respectively. These distances were much higher than the median values obtained among the sequences of the other species of the genus (10.28%) or the values obtained between those collected from the same geographic area (*A. temminckii* and *A. cf. leucostictus* from French Guiana, K2P = 2.21%; *A. spinosus* and *A. chagresi* from Panama, K2P = 3.02%).

### 3.2. Cytogenetic Analysis

**Figure 2:**
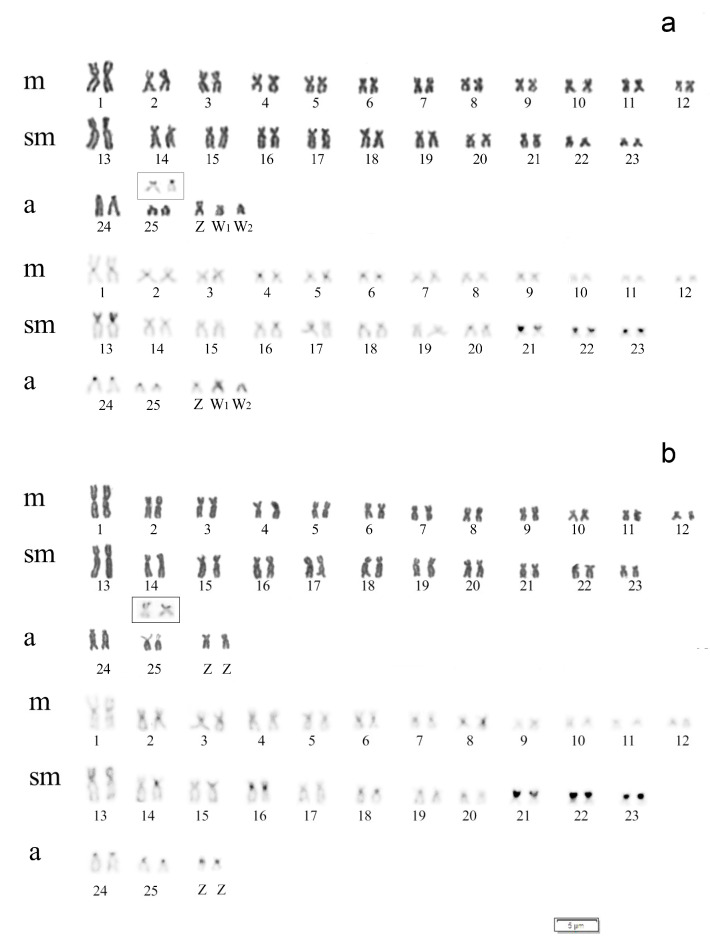
Male (**a**) and female (**b**) *A. clementinae* karyotypes arranged after Giemsa (above) and C-banding (below) staining. The chromosome pair showing Ag-NOR, after silver staining, is shown in the inset. The differences observed in both karyotypes support the occurrence of a multiple ZZ/ZW_1_W_2_-sex chromosome system, with females being the heterogametic sex.

**Figure 3:**
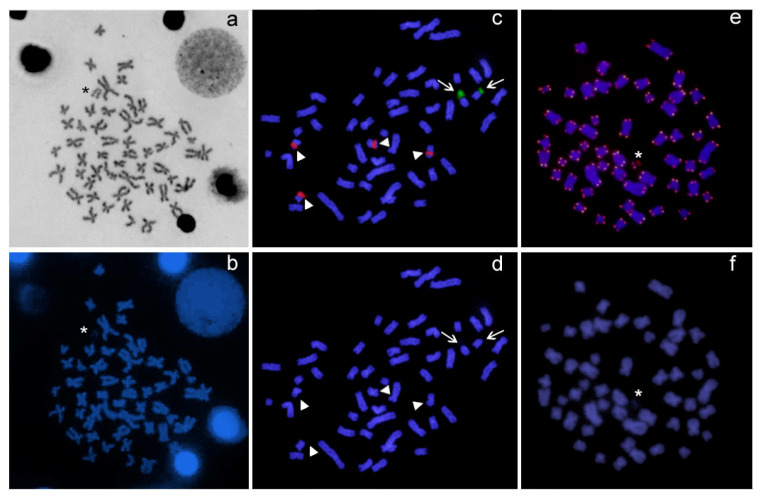
Mitotic chromosomes of *A. clementinae*. Female metaphase in sequential (**a**) Giemsa and (**b**) DAPI staining; male (**c**) after dual-color FISH experiments with ribosomal 18S (green) and 5S (red) rDNA probes and corresponding DAPI (**d**); female metaphases hybridized with the telomeric (TTAGGG)_n_ probe (**e**) and corresponding DAPI (**f**). Asterisk indicates the W_2_ chromosome; arrows indicate 18S rDNA sites and arrowheads 5S rDNA sites.

Differences in chromosome number were observed between specimens of A. clementinae of both sexes. Males showed a diploid number (2n) of 52 and a karyotype composed of 48 m/sm + 4st/a chromosomes (FN = 100), while females showed 2n = 53 and a karyotype composed of 48 m/sm + 5a chromosomes (FN = 101) (Figure 2). The differences observed in these karyotypes evidenced the occurrence of a multiple sex chromosome system of the ZZ/ZW_1_W_2_ type, with females being the heterogametic sex. Sequential Giemsa/DAPI staining revealed the absence of AT-rich chromosome regions, and the presence of a female-exclusive acrocentric chromosome, dark and hardly distinguishable in DAPI, that corresponded to one of the W sex chromosomes, that we indicated as the W_2_ (Figure 3a,b). 

The C-positive heterochromatin blocks were located in the centromeric/pericentromeric regions of almost all chromosomes and on the entirely heterochromatic short arms of chromosomal pairs 13, 21, 22, 23. Moreover, the sex chromosomes W_1_ and W_2_ also presented faint C-positive blocks (Figure 2). Sequential staining revealed that Ag-NOR sites covered the distal region of the short arms on a single pair of chromosomes (pair n. 25) presenting very weakly Giemsa-stained uncondensed chromatin, corresponding to chromosome regions darkly stained after silver staining, i.e., Ag-NORs (Figure 2, inset).

**Figure 4:**
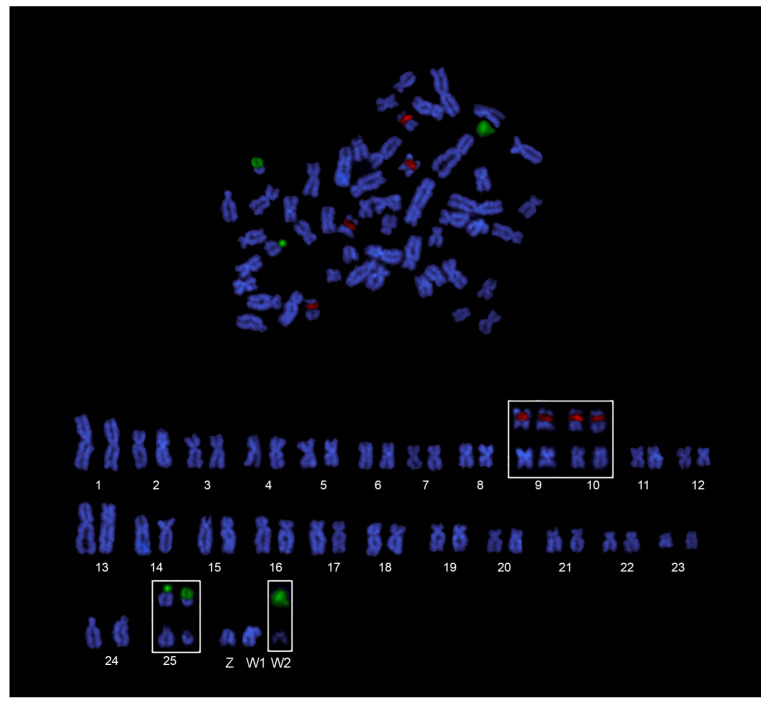
Female dual-color FISH *A. clementinae* metaphase (**above**) and karyotype (**below**) after 18S (**green**) and 5S (**red**) rDNA probes.

The chromosomal mapping of major and minor ribosomal genes (Figure 3c) confirmed the presence of two 18S rDNA sites likely corresponding to NORs, and four 5S rDNA sites, non-syntenic, and located on the short arms of two small metacentric pairs (Figure 4). Females showed an additional 18S rDNA site on the acrocentric DAPI-dark W_2_ chromosome (Figure 4). FISH with the telomeric probes evidenced signals to telomeres of all the chromosomes of the karyotypes and neither interstitial telomeric sites (ITSs), nor differences between males and females, were observed (Figure 3e,f).

## 4. Discussion

The fish examined in this study—the first *Ancistrus* species from the trans-Andean area (Ecuador)—display morphological traits (arrangement of the soft, fleshy tentacles on the snout, number of movable cheek odontodes, shorter dorsal fin and pectoral fin spines), that are diagnostic of *A. clementinae,* as described by Provenzano and Barriga [12]. They all belong to the same exclusive monophyletic group and the *Ancistrus* genus, as determined by molecular analysis and phylogenetic reconstruction, and show high genetic distances when compared with the other species of the genus.

Our cytogenetic findings support the presence of a ZZ/ZW_1_W_2_ sex chromosomal system in fishes and allowed some consideration of its origin/features: (a) the first identification of this system in *Ancistrus* reinforces the picture of plasticity of sex chromosome differentiation among different species of the same genus; (b) this rare multiple sex chromosome system, derived from a simple ZW-system, differentiated through the accumulation of repetitive DNAs; (c) these latter include rDNAs, and a close relationship exists between rDNAs and sex chromosomes differentiation.

The karyological results for this species support the notion that *Ancistrus* has a considerable cytogenetic diversity and exhibits “rapid karyotypic evolution” [42]. Although 2n = 52 chromosomes is compatible with the plesiomorphic karyotype of the tribe Ancistrini [43], this chromosome number is only recorded in a small number of *Ancistrus* species (10/52 records, see [17]), with the rest showing lower values. In addition, the sex chromosome system identified in this species was never detected before in the genus: females of *A. clementinae* (2n = 53) showed an acrocentric chromosome (without a homologous element) that is absent in males (2n = 52), thus suggesting the existence of a♂ZZ/♀ZW_1_W_2_ MSC.

This novel sex chromosomal system raises the question of how fish determine their sex, a puzzling topic that is an intriguing subject for both evolutionary biology and aquaculture practices. Recently, among fishes, the analysis of 440 verified records, showed a variety of chromosomal sex-determination systems ranging from the most common male (♀XX/♂XY) or female (♂ZZ/♀ZW) heterogamety, to variations of these involving loss of the Y or W sex chromosome (♀XX/♂X0, ♂ZZ/♀Z0), and to multiple systems (♀XX/♂XY_1_Y_2_, ♀X_1_X_1_X_2_X_2_/♂X_1_X_2_Y, ♀X_1_X_1_X_2_X_2_/♂X_1_Y_1_X_2_Y_2_, ♂ZZ/♀ZW_1_W_2_, ♂Z_1_Z_1_Z_2_Z_2_/♀Z_1_W_1_Z_2_W_2_), usually derived from chromosomal fusions and/or fissions [44]. These latter were reported in 75 cases, 80% of which likely originated independently [44]. Among teleosts, which are characterized by a frequent turnover of sex chromosomes, the main mechanisms giving rise to such multiple systems are sex chromosome–autosome fusions. They occur more frequently in male-heterogametic than in female-heterogametic taxa and can be influenced by the interaction between fish populations, differing in their sex chromosomes [44]. Unlike the progressive suppression of recombination that often occurs in simple sex chromosome systems, this suppression may be accomplished immediately by the chromosomal rearrangements that gave rise to the MSC [45]. This dynamic condition is often linked to, and likely driven by, the presence of repetitive sequences, that can also promote other rearrangements, such as chromosome fissions, which were also suggested to be the origin of MSC in other Loricariidae species [17]. As the location of the sex-determining region has not been identified yet, the assignation of W_1_ (ancestral) and W_2_ (neo) sex chromosomes in different species was performed arbitrarily. Indeed, in the Loricariidae, these chromosomes typically have highly similar morphologies, making it challenging for their precise identification and their correct assemblage [46].

The genome composition analysis and chromosome mapping revealed that fish sex chromosomes are typically enriched with repetitive sequences involved in their heterochromatinization and differentiation. The most common sequences found in their genomes include satellite DNAs, 18S and 5S rDNAs, transposable elements (TEs) and microsatellites [45,47]. However, usually, multiple sex chromosomes are not characterized by heterochromatin accumulation [48,49], and can show ITSs [50,51]. 

Both W_1_ and W_2_ sex chromosomes of *A. clementinae* are characterized by heterochromatin accumulation, according to what was detected in other species of the genus showing XX/XY [23,52] and ZZ/ZW sex chromosomes [53,54]. However, this is not a general rule, as such accumulation was not observed in other *Ancistrus* species that also present simple or multiple chromosome systems [21,22]. In addition, the W_2_ chromosome is enriched with 18S rDNA repeats, as evident after the in situ hybridization with a major ribosomal genes probe (but not with silver staining). DAPI faint-staining of this chromosome, both as FISH counterstaining and as sequential staining after Giemsa, clearly indicated that this chromatin is AT-poor [55], according to the general (but not absolute) rule that ribosomal chromatin is GC-rich [56]. 

The presence of ribosomal repeats on this chromosome is remarkable, as the occurrence of a single chromosome pair bearing major ribosomal genes in a terminal position along chromosome arms is the most common characteristic observed among teleost fishes [56,57]. In *Ancistrus,* (where a single pair of major rDNA sites is reported in 51 out of 52 records, see [17]), these sequences were not detected before on sex chromosomes, although present in other loricariids, such as *Harttia* [46,58] and *Rineloricaria* [49]. On the contrary, a strong association between ribosomal genes and sex chromosomes was reported for Eleotridae [59] and Triportheidae [60,61,62,63], including all the species of the genus *Triportheus.* These latter are all characterized by the presence of a ZW-type sex-determination system, where the W chromosome shows large heterochromatic blocks and the accumulation of a huge 18S rDNA block. These ribosomal sequences were considered to play a role in reducing recombination between sex chromosomes and, thus, in their evolution [45], according to the idea that rDNAs facilitate Robertsonian fusions and, therefore, also promote karyotype evolution [64]. In *A. clementinae,* the rDNA cluster present on the W_2_ chromosome is inactive and this could be due to dosage compensation, as observed for sex-linked rDNA genes in turtles, where complex mechanisms of upregulation or silencing exist, likely mediated by retrotransposon [65]. 

Other repetitive sequences were used for FISH experiments to check whether they are mapped on sex chromosomes in *Ancistrus.* The results showed that X chromosomes (in the XX/XY system) are enriched with microsatellite repeats [22]. On the other hand, both simple and multiple sex chromosome systems lack accumulation of the retro-TEs of the Rex family [42], sequences that are widely distributed in the genomes of other fishes [66], including a putative proto-sex chromosome [67]. The next steps must include the mapping of these repeated sequences in *A. clementinae* to clarify whether they are associated with the heterochromatinization of W_1_ and W_2_ chromosomes.

Minor ribosomal genes, on the contrary, usually, are present in multiple chromosome sites in *Ancistrus,* and this pattern was confirmed in *A. clementinae.* In many species of Ancistrini, the 5S rDNA genes are syntenic with those of 18S rDNA [42,43,68,69]. The multiple 5S rDNA sites, due to their intensive activity and chromatin decondensation, have been proposed to act as hotspots of chromosome rearrangements (breakpoint regions for the fusion) in Loricariidae and *Ancistrus* [70,71,72,73,74]. However, it remains debated whether the dispersion of these ribosomal sequences in fish genomes is linked to the presence of TEs and is a byproduct of genome/chromosome re-arrangements [75]. The potential effects of the accumulation of repetitive DNA and its influence on the frequency of recombination have been shown in many fish species [e.g. 17,19,21,46,58,60–62], which may help to explain how it first accumulated on the sex chromosomes. Repetitive sequences should rapidly accumulate once the sex chromosomes stop recombining [76]. Following the theory of concerted evolution, the evolution of rDNA sequences preserves the functionality and homogeneity of these genes [77,78]. However, it is possible that unequal sister chromatid recombination or retro-transposition lead to favoring of a copy number variation of such sequences [57,78]. The non-transcribed rDNA copies that can result from this copy number variation are extremely important for maintaining the integrity of the genome and are extensively reported on fish genomes [57].

Although the mechanisms of origin and possible differentiation of the ZW_1_W_2_ MSC present in *A. clementinae* cannot be fully resolved by the current data, the remarkable and ongoing chromosomal divergence in *Ancistrus* is confirmed by the findings of this investigation. It is important to note that the case herein described represents the third fish species harboring a ZZ/ZW_1_W_2_ MSC and the first Loricariidae [44], which, given the scarcity of cytogenetic data in *Ancistrus* and other species of the family, suggests that additional cases may be discovered. More studies are required to determine the origin of such MSC and its potential role in species diversification. Future research combining molecular phylogenetics and cytogenetic approaches is needed on other congeneric species from Ecuador in order to describe new karyotypes, assign them to certain identified taxa, and, if necessary, reconstruct a phylogeographical pattern. Previous data, almost entirely from Brazil, showed that chromosome rearrangements and plasticity in this genus do not seem to follow a geographic pattern. In order to fully understand the long-term effects of historical hydrogeological dynamics events in isolating populations/species of these fishes, other endemic Loricariidae species that are part of the same biotic community, such as *Transancistrus santarosensis* and *Chaetostoma bifurcum*, should also be considered in the analysis. In addition, a robust phylogenetic reconstruction that includes all *Ancistrus* karyotyped to date could provide insights into the chromosome evolution of such an interesting fish model.

## 5. Conclusions

Chromosomal data on *A. clementinae* here reported allowed us to add new information on Ancistrini karyology and identify a new case of multiple sex chromosomes, rare among fishes. This karyotype corresponds to the ancestral one recognized for the tribe and the genus, and the species is basal within *Ancistrus* phylogenetic tree. Future research should clarify if Robertsonian fusions that allowed 2n reduction and thus chromosomal evolution in the genus and repeated sequences dynamics follow the same evolution pattern and are congruent with species divergence as disclosed by molecular phylogenetic analysis. 

## Data Availability

Sequences are deposited in GenBank (A.N. OQ132522-23).

## Supplementary Materials

The following supporting information can be downloaded at: https://www.mdpi.com/article/10.3390/genes14020306/s1, Figure S1: Neighbour-joining tree based on *Ancistrus cytochrome* oxidase I gene (COI) sequences; Table S1: *Ancistrus clementinae.* Summary of the information on collection date and site, sex and analysis of the specimens; Table S2: GenBank Accession Number and sampling area of *Ancistrus* speices and of the outgroup (*Lithoxus stocki*) COI sequences used in phylogenetic analyses.
