# Supplementary material for: Occurrence of Sex Chromosomes in Fish of the Genus Ancistrus with a New Description of Multiple Sex Chromosomes in the Ecuadorian Endemic Ancistrus clementinae (Loricariidae)
Source: Genes (Basel). 2023 Jan 24;14(2):306. doi: 10.3390/genes14020306 (PMC9956960; doi:10.3390/genes14020306)
Supplement: Supplementary file 1 [file genes-14-00306-s001.zip › Nirchio Supplementary Table2.pdf]

Table S2. GenBank Accession Number and sampling area of *Ancistrus* species and of the outgroup (*Lithoxus stocki*) COI sequences used in phylogenetic analyses

**Species**	**A.N.**	**Sampling area**
A. aguaboensis	MK464024	Brazil, Ribeirão Cafuringa
A. brevipinnis	EU359402	not available
A. brevipinnis	MG825021	Brazil, Rio Grande do Sul
A. chagresi	MG936656	Panama, Rio Cocle del Norte
A. cirrhosus	GU701863	Brazil, Upper Parana Basin
A. cirrhosus	GU701865	Brazil, Upper Parana Basin
A. cryptophthalmus	JX477635	not available
A. cryptophthalmus	JX477637	not available
A. cryptophthalmus	JX477641	not available
A. dolichopterus	KP772578	Brazil, Nhamundá River
A. dolichopterus	KP772593	Brazil, Nhamundá River
A. cf. leucostictus	MZ051870	French Guiana, Maroni River
A. cf. leucostictus	MZ051872	French Guiana, Litany River
A. cf. leucostictus	MZ051912	French Guiana, Maroni River
A. multispinis	MG825030	Brazil, Rio de Janeiro
A. spinosus	MG936657	Panama, Rio Tuira
A. temminckii	MZ051822	French Guiana, Maroni River
A. temminckii	MZ051978	French Guiana, Maroni River
A. temminckii	MZ052035	French Guiana, Maroni River
Lithoxus stocki	MZ051988	Fench Guiana, Maroni River
