# Supplementary material for: Occurrence of Sex Chromosomes in Fish of the Genus Ancistrus with a New Description of Multiple Sex Chromosomes in the Ecuadorian Endemic Ancistrus clementinae (Loricariidae)
Source: Genes (Basel). 2023 Jan 24;14(2):306. doi: 10.3390/genes14020306 (PMC9956960; doi:10.3390/genes14020306)
Supplement: Supplementary file 1 [file genes-14-00306-s001.zip › Nirchio et al_Table S1.pdf]

Table S1 *Ancistrus clementinae*. Summary of the information on collection date and site, sex and analysis of the specimens

**Individual**	**Sex**	**Date of collection**	**Collection site**	**COI sequence**	**Cytogenetics**
UTMACH-0131	M	17/10/2015	Río Palenque, Cantón Pasaje		X
UTMACH-0132	F	05/11/2015	Río Palenque, Cantón Pasaje		X
UTMACH-0133	M	05/11/2015	Río Palenque, Cantón Pasaje	X	X
UTMACH-0134	M	11/11/2015	Río Palenque, Cantón Pasaje		X
UTMACH-0135	F	11/11/2015	Río Palenque, Cantón Pasaje	X	
UTMACH-0136	F	12/11/2015	Río Palenque, Cantón Pasaje		
UTMACH-0153	F	18/02/2016	Río Palenque, Cantón Pasaje		X
UTMACH-0154	M	18/02/2016	Río Palenque, Cantón Pasaje	X	
UTMACH-0164	F	01/03/2016	Río Palenque, Cantón Pasaje		X
UTMACH-0238	F	28/06/2017	Río Palenque, Cantón Pasaje		X
UTMACH-0239	M	28/06/2017	Río Palenque, Cantón Pasaje	X	X
UTMACH-0243	F	28/06/2017	Río Palenque, Cantón Pasaje		X
UTMACH-0245	F	28/06/2017	Río Palenque, Cantón Pasaje	X	X
UTMACH-0246	F	10/07/2016	Río Palenque, Cantón Pasaje	X	X
UTMACH-0247	F	10/07/2016	Río Palenque, Cantón Pasaje	X	X
UTMACH-0248	F	10/07/2016	Río Palenque, Cantón Pasaje	X	X
UTMACH-0249	F	10/07/2016	Río Palenque, Cantón Pasaje	X	X
UTMACH-0415	M	27/07/2021	Río La Moquillada, Cantón Las Lajas	X	X
UTMACH-0416	F	27/07/2021	Río La Moquillada, Cantón Las Lajas	X	X
UTMACH-0417	M	29/07/2021	Río La Moquillada, Cantón Las Lajas	X	X
UTMACH-0418	F	29/07/2021	Río La Moquillada, Cantón Las Lajas	X	X
UTMACH-0419	M	30/07/2021	Río La Moquillada, Cantón Las Lajas	X	X
UTMACH-0420	M	10/08/2021	Río La Moquillada, Cantón Las Lajas	X	X
UTMACH-0421	M	13/08/2021	Río Palenque, Cantón Pasaje		X
