# Supplementary figures and images for: Occurrence of Sex Chromosomes in Fish of the Genus Ancistrus with a New Description of Multiple Sex Chromosomes in the Ecuadorian Endemic Ancistrus clementinae (Loricariidae)
Source: Genes (Basel). 2023 Jan 24;14(2):306. doi: 10.3390/genes14020306 (PMC9956960; doi:10.3390/genes14020306)

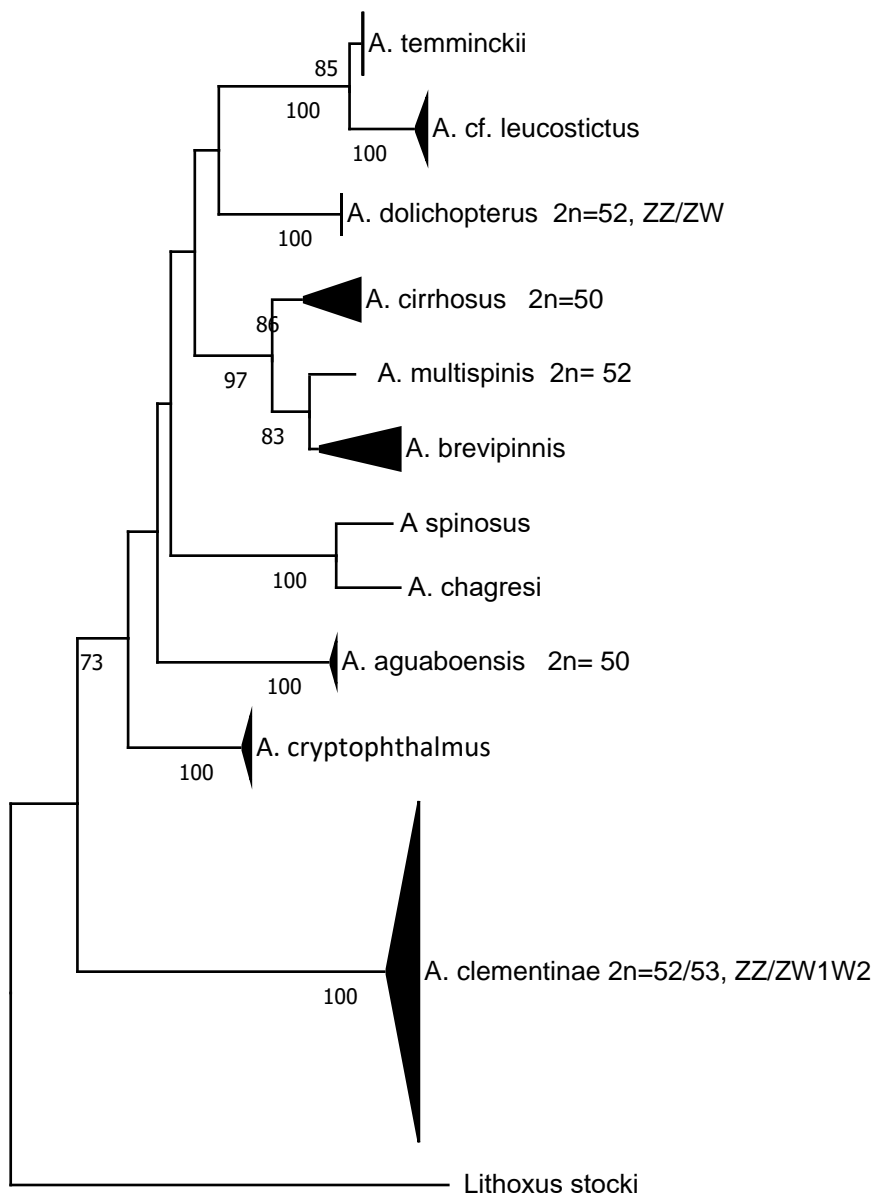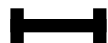

0.02

Supplement: Supplementary file 1 [file genes-14-00306-s001.zip › Nirchio et al Fig S1.pdf]
